# Enzymatic cleavage of histone H3: a new consideration when measuring histone modifications in human samples

**DOI:** 10.1186/s13148-014-0041-5

**Published:** 2015-01-22

**Authors:** Caitlin G Howe, Mary V Gamble

**Affiliations:** Department of Environmental Health Sciences, Mailman School of Public Health, Columbia University, 650 W. 168th Street, Room 1618, New York, NY 10032 USA; Department of Environmental Health Sciences, Mailman School of Public Health, Columbia University, 722 W. 168th Street, Room 1107E, New York, NY 10032 USA

**Keywords:** Histone H3, Enzymatic cleavage, Histone modifications

## Abstract

Histone modifications are increasingly being used as biomarkers of cancer prognosis and survival. However, we identified a cleavage product of histone H3 in human peripheral blood mononuclear cells, which interferes with measures of certain H3 modifications. Therefore, the potential for enzymatic cleavage of histones should be considered when measuring histone modifications in human samples. Furthermore, the enzymatic cleavage of human H3 is itself a fascinating area of research and two important questions remain to be answered: 1) Does cleavage of human H3 occur *in vivo*, as it does in other organisms? and 2) Does it serve a biologically important function?

## Enzymatic cleavage of histones

Histone modifications are increasingly being used as biomarkers of cancer prognosis [1]. However, histones are very sensitive to enzymatic degradation by proteases [2], and there is evidence from many organisms that histones are enzymatically cleaved *in vivo*; this topic is receiving increasing attention and has been reviewed recently by several groups [3-5]. Enzymatic cleavage of H3 has been observed in tetrahymena [6], yeast [7,8], chicken [9], quail [10], and mouse [11,12]. Furthermore, certain viruses can cleave host cell H3 [13,14], and antimicrobial peptides derived from the N-terminal region of various histones (e.g., H2A, H2B, H1) have been identified in several organisms, including fish [15-20], molluscs [21,22], frogs [23], and even from the gastrointestinal tract [24] and wound fluids [25] of humans.

**Figure 1:**
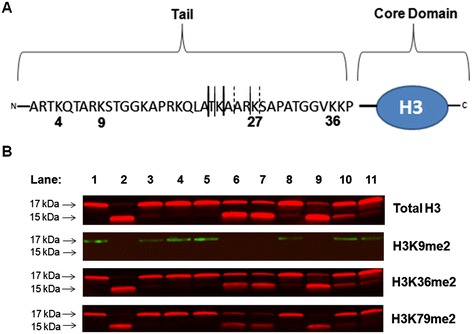
**Enzymatic cleavage of H3 interferes with the measurement of certain histone modifications. (A)** Known enzymatic cleavage sites in H3 for mouse ESCs [11]. Bold solid lines indicate sites that are frequently cleaved, thin solid lines indicate sites that are less frequently cleaved, and dotted lines indicate sites that are rarely cleaved [11]. **(B)** Western blot (Odyssey® CLx Infrared Imaging System, Li-Cor) was used to measure total H3 protein levels (Sigma, H0164, 1:4,000) in 11 representative histone samples that had been isolated, using an acid-extraction method [27], from PBMCs collected from arsenic-exposed Bangladeshi adults enrolled in the Folic Acid and Creatine Trial (FACT), a randomized controlled trial of folic acid and creatine supplementation; sample collection and processing for this study has been described previously [28]. The expected size of H3 is ~17 kDa. A distinct cleavage product of H3 is observed at ~15 kDa, and an additional H3 cleavage product between 15 and 17 kDa is also present in several of the samples (top panel). In the same 11 samples, three histone modifications that are located in different regions of H3 were assessed by Western blot: H3K9me2 (Abcam, ab1220, 1:1,000, mouse) (second panel), H3K36me2 (Abcam, ab9049, 1:1,000, rabbit) (third panel), and H3K79me2 (Abcam, ab3594, 1:400, rabbit) (fourth panel).

**Figure 2:**
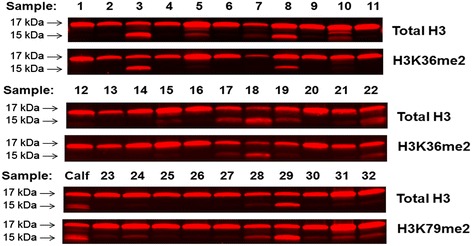
**Extensive H3 cleavage is evident in approximately one-third of PBMC histone samples, but it does not affect measures of H3K36me2 and H3K79me2.** Total H3 was measured in an additional 32 histone PBMC samples from the FACT study and in histones from calf thymus (Sigma-Aldrich). H3K36me2 was also measured in 22 of the PBMC samples (Samples 1–22), and H3K79me2 was measured in calf histones and in ten of the PBMC samples (Samples 23–32).

Until recently, there were few reports of histone cleavage in human cells. However, last year, Vossaert et al. reported histone H3 clipping in human embryonic stem cell (ESC) lines [26], and our group recently identified a cleavage product of H3 in human peripheral blood mononuclear cells (PBMCs) (Figure 1). We observe this H3 cleavage product in spite of the use of protease inhibitors during histone isolation, including a protease inhibitor cocktail (Roche), which inhibits enzymatic cleavage of H3 in human ESCs [26], and E-64, which inhibits cathepsins, including Cathepsin L, which cleaves H3 in mouse ESCs [11]. The H3 cleavage product that we observe in human PBMCs is similar in size to the H3 cleavage product observed in mouse ESCs [11]. Extensive cleavage of H3 is observed in approximately one-third of these PBMC histone samples (Figure 2).

Based on Western blot, we have determined that H3 cleavage interferes with the measurement of certain histone modifications. Figure 1A illustrates the known enzymatic cleavage sites in H3 for mouse ESCs [19]. In Figure 1B, Western blots illustrate total H3 (top panel) with varying degrees of histone cleavage for 11 representative PBMC histone samples that were collected from participants enrolled in the Folic Acid and Creatine Trial (FACT), a randomized controlled trial of folic acid and creatine supplementation in Bangladeshi adults [28]. Figure 1B also shows, for the same 11 PBMC samples, three histone modifications that vary in relation to their location on histone H3 (i.e., upstream or downstream of the cleavage sites shown in Figure 1A). For example, Figure 1B illustrates H3K9me2 (second panel), a modification located downstream of known H3 cleavage sites. Samples without large amounts of H3 cleavage (Lanes 1, 3–5, 8, 10, 11) have detectable H3K9me2. In contrast, samples with extensive cleavage of H3 (Lanes 2, 6, 7, 9) have no detectable H3K9me2. Figure 1B also illustrates H3K36me2 (third panel) and H3K79me2 (fourth panel), which are histone modifications located upstream of H3 enzymatic cleavage sites; H3K36me2 is located in the tail region of H3, and H3K79me2 is located in the core domain of H3 (Figure 1A). H3K36me2 and H3K79me2 can be detected both in the 17-kDa band of H3 that has not been cleaved and in the <17-kDa bands of H3 that have been cleaved (Figures 1B and 2). H3 cleavage is also detectable in histones from calf thymus (Figure 2). This has been described previously by several groups [3,29]. Similarly, cleavage of calf thymus H3 does not interfere with upstream histone modifications, such as H3K79me2 (Figure 2). Collectively, these data suggest that H3 cleavage only influences the ability to detect histone modifications that are situated downstream of histone cleavage sites.

## Implications for molecular epidemiology studies

Since it is unclear when enzymatic cleavage of H3 occurs in human PBMC samples, it is difficult to know if it is of biological or methodological interest. Regardless, enzymatic cleavage of H3 has important implications for measuring global histone modifications in human samples. Currently, the most commonly studied histone modifications include methylation and acetylation marks on H3K4, H3K9, and H3K27, which all fall within the N-terminal tail region of H3. However, the portion of H3 that is clipped off in mouse and human cells includes these residues. Thus, measures of marks on H3K4, H3K9, and H3K27 may be underestimated if samples have experienced enzymatic cleavage of H3; this is particularly true for antibody-based methods, such as ELISA and immunohistochemistry methods, which cannot take into account H3 cleavage. Modifications that lie on amino acid residues upstream of H3 enzymatic cleavage sites, such as H3K36 and H3K79, do not appear to be affected by the enzymatic cleavage of H3 and therefore can be measured accurately, regardless of cleavage.

A better understanding of when and why enzymatic cleavage of H3 occurs is essential. If enzymatic cleavage of human H3 occurs *in vivo*, this may be an important biological phenomenon. Alternatively, if enzymatic cleavage occurs as a result of sample collection and processing, preventive measures must be developed such that all global histone modifications on H3, including modifications on H3K4, H3K9, and H3K27, can be accurately measured. In the meantime, for banked samples previously collected for the measurement of global histone marks, Western blot can be used to check samples for enzymatic cleavage of histones. If histone cleavage products are observed in samples, it may not be appropriate to measure certain histone marks based on their location.

## Abbreviations

ESCs: Embryonic stem cells
PBMCs: Peripheral blood mononuclear cells
